# Clinical wear of approximal glass ionomer restorations protected with a nanofilled self-adhesive light-cured protective coating

**DOI:** 10.1590/1678-7757-2018-0094

**Published:** 2018-10-02

**Authors:** Daniela Hesse, Clarissa Calil Bonifácio, Cornelis Johannes Kleverlaan, Daniela Prócida Raggio

**Affiliations:** 1 Academic Centre for Dentistry Amsterdam, Department of Cariology, Pedodontology and Endodontology, Amsterdam, The Netherlands; Universidade de São Paulo, Faculdade de Odontologia, Departamento de Ortodontia e Odontopediatria, São Paulo, São Paulo, Brasil.; Academic Centre for Dentistry Amsterdam, Department of Cariology, Pedodontology and Endodontology, Amsterdam, The Netherlands; Universidade de São Paulo, Faculdade de Odontologia, Departamento de Ortodontia e Odontopediatria, São Paulo, São Paulo, Brasil.; 2 Academic Centre for Dentistry Amsterdam, Department of Cariology, Pedodontology and Endodontology, Amsterdam, The Netherlands.; 3 Academic Centre for Dentistry Amsterdam, Department of Dental Materials Science, Amsterdam, The Netherlands.; 4 Universidade de São Paulo, Faculdade de Odontologia, Departamento de Ortodontia e Odontopediatria, São Paulo, São Paulo, Brasil.

**Keywords:** Dental restoration wear, Glass ionomer cements, Deciduous tooth

## Abstract

High viscous glass ionomer cement (GIC) has gained popularity as a restorative material; however, high wear is pointed as one of the major drawbacks of this material. Protective surface coatings were developed to protect GIC from water contamination with the additional advantage of occluding any surface cracks or porosities commonly found in this material, possibly resulting in an increased wear resistance of the restorations.

**Objective:**

The objective of this study was to investigate the clinical wear of GIC approximal restorations in primary molars protected either with a nanofilled self-adhesive light-cured protective coating (NPC) or with petroleum jelly.

**Material and Methods:**

Approximal caries lesions in primary molars from 32 schoolchildren previously enrolled in another clinical trial were included in this investigation. GIC restorations were performed according to the Atraumatic Restorative Treatment approach and protected with either petroleum jelly or a NPC. Impressions of the restored hemiarch were done after 1 day and 6, 12, 24 and 36 months. The impressions were scanned in a 3-D appliance and the obtained images were superimposed using an appropriate computer software. Two-way ANOVA for repeated measures and Tukey's post-hoc test were used to analyze the wear of restorations (α=5%).

**Results:**

A significant difference was found between the two groups, with a wear protection offered by the application of a NPC. Conclusion: These results suggest that the application of a NPC has a protective effect on the clinical wear of approximal GIC restorations in primary teeth.

## Introduction

High viscous glass ionomer cement (GIC) has gained popularity in contemporary dentistry, especially in pediatric dentistry, where this material is considered a viable option to restore dental caries lesions. ^3^ The main advantages attributed to this material are the ease of use due to its bulk application and acceptable physical-mechanical properties, the last property is a result from its high powder/liquid ratio (3:1 to 4:1). Additionally, the GIC presents good biocompatibility and chemical adhesion to tooth structures, ^3^ ^,^ ^32^ as well as the ability of fluoride release and recharge with external sources, which is believed to benefit the restored tooth, ^26^ ^,^ ^32^ and possibly the surrounding restored surfaces, ^30^ which could be considered an advantage, especially for high caries-risk patients.

However, GIC presents some peculiarities that must be respected to ensure its best properties. Essentially, high viscous GIC is composed of a basic (ionleachable) aluminosilicate glass powder that is mixed with an aqueous solution of polymeric acid, resulting in a viscous paste. The setting of GIC occurs in the presence of water, as the polymeric acids needs this medium to releases protons, therefore, starting the acid reaction and setting of the material. During this reaction, the hydrated protons of the acid interact with the glass particles and release metal ions. As a result, some soluble salts such as calcium polyacrylates are formed and gradually replaced by insoluble aluminum polyacrylate salts, leading to the hardening of the cement. This process occurs 24 h after the material's mixing and during this period the GIC is sensitive to water exchanges, which may interfere in the material's mechanical properties. ^27^ If prematurely exposed to moisture, it may lose substance, which is clinically perceived as surface wear and reduced translucency. If the setting reaction happens in a dry environment, the GIC tends to lose water, which results in dimensional changes, adhesion problems and the formation of internal cracks, reducing the material's strength. ^20^ ^,^ ^28^

Some materials are indicated to overcome the issues related to water sensitivity and protect the GIC surface. Varnishes, adhesive systems and petroleum jelly can be used as GIC coatings and among those, petroleum jelly is considered a good option due to its safety and biocompatibility; ^7^ however, petroleum jelly can be easily washed away ^13^ and a surface coating that lasts longer is desired, as the GIC should be isolated from moisture during the entire setting period. Given this context, a new generation of nanofilled self-adhesive light-cured protective coating (NPC) for GIC was developed, claiming to isolate the GIC from saliva contamination during the complete maturation of the material, as well as occluding any surface cracks and porosity of the GIC, reinforcing the strength of this material and therefore, increasing its wear resistance, ^17^ without compromising the fluoride release when compared to petroleum jelly application ^21^ nor the caries-preventive effect of GIC. ^31^

Some laboratory studies have simulated the wear of specimens protected with this NPC and contradictory results were found. ^6^ ^,^ ^24^ However, investigating the clinical wear of GIC coated restorations is relevant, as the dental material is exposed to the intraoral environment, in which a complex process involving masticatory loading in the presence of a chemically active environment contributes to the degradation of the restoration. ^12^ Attempting to answer this question, a clinical trial was performed to evaluate the clinical wear of GIC occlusal restorations in permanent molars with and without the application of a NPC; ^11^ however this investigation assessed the wear of restorations in permanent molars and through clinical evaluation, pictures and visual assessment of the casts obtained from the restorations. To date, no published studies performed a quantitative analysis of the clinical wear of coated GIC approximal restorations in primary molars through digital analysis of subtracted scanned images, which could help to explain the behavior of GIC restorations after long-term exposure to the masticatory load. Thus, the objective of this study was to investigate the clinical wear of approximal GIC restorations in primary molars protected with a NPC compared to restorations protected with petroleum jelly. The hypothesis tested was that the application of a NPC would result in decreased wear of GIC when compared to the protection offered by petroleum jelly.

## Material and methods

### Study design

This is an *in vitro* investigation that is nested to another randomized controlled trial with a parallel group design ^19^ that was performed in the state of São Paulo, Brazil and was registered in a virtual platform for registration of experimental studies on humans (Registro Brasileiro de Ensaios Clínicos #RBR-2nwk89). This study was approved by the local Research Ethics Committee (University of São Paulo #190/08) and the parents or legal guardians gave consent before starting the trial through an informed consent form.

### Clinical procedures

The main study consisted of four protocols for treating primary molars according to the Atraumatic Restorative Treatment (ART) premises. The implementation of the study has been described in detail in another study. ^19^ Shortly, 208 children (6-7 years old) that presented approximal cavitated caries lesions were included in the main study. Only one cavity *per* child was selected and four trained operators were responsible for conducting the operative phase in the main study. ^19^

**Figure 1:**
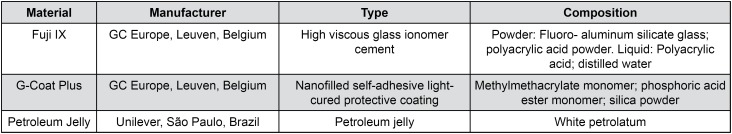
Materials used in the study

In this study, a total of 32 restorations were evaluated. The treatments were performed according to the ART guidelines ^15^ and the cavities were restored using the high viscous GIC Fuji IX (GC Europe, Leuven, VB, Belgium). After inserting the GIC in the cavity, the press-finger technique using a thin layer of petroleum jelly was performed in both groups. Half the sample had the surface protected by the application of a thin layer of petroleum jelly (Unilever, São Paulo, SP, Brazil - n = 16) and the other half by the application of a NPC (G-Coat Plus, GC Europe, Leuven, VB, Belgium - n=16). In the petroleum jelly group, the occlusion was checked and the GIC excess was removed using hand instruments. After the initial setting of the GIC, a new layer of petroleum jelly was applied to protect the material. In the NPC group, the occlusion was checked and the excess of GIC was removed using hand instruments. After that, new cotton rolls were placed to minimize the contamination effect of saliva and the petroleum jelly was completely removed from the restoration surface using gauze pads. Then, the NPC was applied with a microbrush on the restoration's occlusal and proximal surfaces. A new matrix band and wedge were applied and the coat was light-cured for 20 seconds on occlusal surfaces, 20 seconds on buccal surfaces, and 20 seconds on lingual surfaces. The materials used in this investigation are shown in Figure 1 .

### Evaluations of restorations

One day after restoration procedure (“baseline”) the patients were recalled and “triple tray” impressions of the hemiarch containing the restoration were taken using an addition-cured silicone (Futura AD - DFL, Rio de Janeiro, RJ, Brazil) and epoxy dies (Arazyn 1.0 - Redelease, São Paulo, SP, Brazil) were obtained for the assessment of the wear of restorations. The patients were recalled after 6, 12, 24 and 36 months and new impressions were made.

### Quantitative wear measurement

The clinical wear of restorations was evaluated using a laser scanner technique by superimposing the scanned images of the epoxy dies in different moments. ^29^ For such, the baseline scanned image was compared to the scanned images of the different evaluated moments. The analysis only included complete impression sets, i.e., only the patients who had the baseline, 6, 12, 24 and 36 months epoxy dies. Since the replica dies were all made of a transparent epoxy resin, they needed to be coated with titanium dioxide powder (3M ESPE, Saint Paul, MN, USA) that was sprayed onto the replicas from a distance of 30 cm. After that, the replica dies were scanned using the Lava™ Chairside Oral Scanner C.O.S. (3M ESPE, Saint Paul, MN, USA). To quantitatively investigate the wear of restorations during the course of three years, the scanned images of the replicas were superimposed using the software Geomagic studio 12 (3D Systems, Rock Hill, SC, USA). The baseline replica image was superimposed with each of the follow-up images (6, 12, 24 and 36 months) and the wear was calculated by comparing the StereoLithography files of the superimposed images. The wear measurement of each patient was calculated considering two thresholds: first, the wear of the entire hemiarch where the restoration was performed was calculated. Then, the 1 mm^2^ area of the restoration that presented the highest wear throughout the 36-months evaluation was selected and the wear of this 1 mm^2^ area was calculated. To perform this second measurement, the software auto-generated a virtual circle of 1.1 mm diameter. Then, the software automatically positioned this circle on the same area of the restorations on the superimposed images corresponding to the 6, 12 and 24 months evaluations. All the procedures were performed by a single researcher, who was blind regarding the groups.

### Statistical analysis

Two-way ANOVA for repeated measures and Tukey's *post-hoc* test were used to test differences in wear measurements of the GIC protected with petroleum jelly or with a NPC and the effect of time/surface protection. A chi-square test was applied to analyze the distribution of patients in both groups regarding the evaluation frames. The data were analyzed with the SPSS 21 software (IBM SPSS, Chicago, IL, USA) using a 95% confidence interval.

## Results

From the original 32 restorations included in this study, 20 were available during the three-year evaluation period: 9 restorations protected with petroleum jelly and 11 with a NPC. Twelve of the original restorations could not be evaluated during the three-year assessment period because the restoration had failed (n=5), the tooth had shaded (n=3), the child was absent from school on the days of the evaluation, had left school or moved away (n=4). The chi-square test showed a similar distribution in both groups regarding the patients lost to follow-up (p>0.05). Table 1 shows the distribution of patients throughout the clinical trial according to surface protection, as well as the reasons of failures and drop-outs recorded in each evaluation time frame.

**Table 1:** Distribution of patients over the clinical trial according to surface protection and reasons for failures/drop-outs

Restoration	Baseline	6 months	Failures/drop-outs	12 months	Failures/drop-outs	24 months	Failures/drop-outs	36 months	Failures/dropouts
	N	N (%)	N (reason)	N (%)	N (reason)	N (%)	N (reason)	N (%)	N (reason)
GIC + petroleum jelly	16	15	1 (bulk fracture of restoration)	10	2 (patients moved)	10	0	9	1 (tooth was absent due to natural exfoliation)
3 (bulk fracture of restoration)									
GIC + NPC	16	15	1 (Inflammation of the pulp)	13	2 (patients moved)	12	1 (tooth was absent due to natural exfoliation)	11	1 (tooth was absent due to natural exfoliation)
Total	32	30	2	23	7	22	1	20	2

Chi-square test showed an equal distribution in both groups regarding the evaluations frames (p>0.05) GIC=glass ionomer cement; N=number of GIC restorations; NPC=nanofilled self-adhesive light-cured protective coating

**Figure 2:**
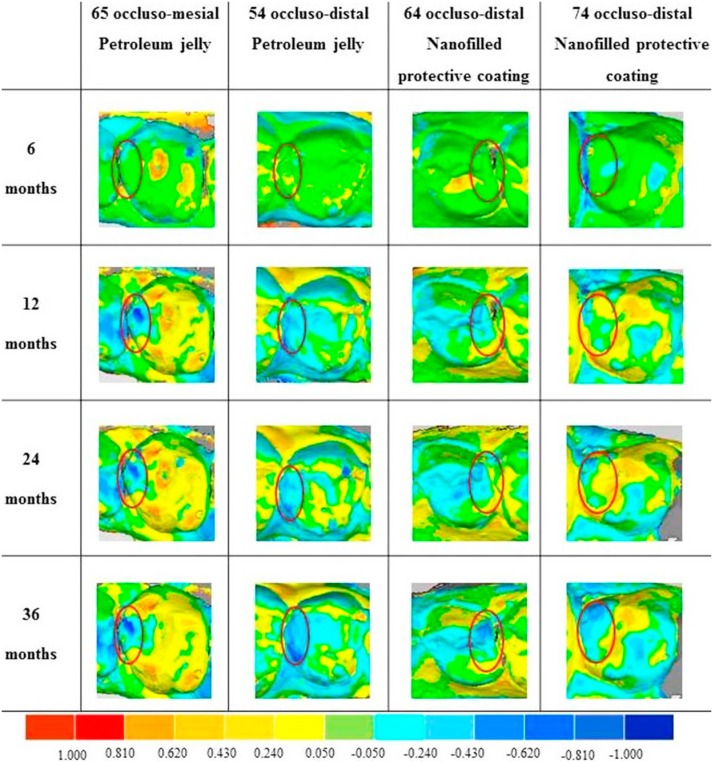
Representative images of the wear of restorations protected with petroleum jelly and a nanofilled self-adhesive light-cured protective coating measured after 6, 12, 24 and 36 months (from top to bottom), respectively. The red circle indicates the area where the restoration was placed. Color bar indicates surface wear (mm). From the right to the left, it is observed that shades of red and yellow indicate decreased wear of the surface, while increased wear of the surface is represented as darker shades of blue

Figure 2 shows representative images of the wear of restorations measured after 6, 12, 24 and 36 months. Yellow and green colors indicate decreased wear of the restoration. Increased wear of restoration is shown as darker shades of blue.

Table 2 presents the mean wear and the standard deviation of the selected 1 mm^2^ area of the restorations and hemiarches. The evaluation of clinical wear of restorations showed a significant difference between both groups when the selected 1 mm^2^ area of the restoration was investigated (p<0.05), with protection offered by the application of a NPC; however, when comparing the entire hemiarch of patients, the difference was not significant (p>0.05).

**Table 2:** Mean wear and the standard deviation in parentheses (µm) of the hemiarch and selected 1 mm^2^ area of the restorations

**Selected area of 1 mm^2^**		
**Analysis time**	**GIC + Petroleum jelly**	**GIC + NPC**
6 months	535 (91)^a,A^	315 (134)^b,A^
12 months	600 (98)^a,A^	364 (135)^b,A^
24 months	610 (93)^a,A^	370 (142)^b,A^
36 months	630 (82)^a,A^	440 (118)^b,A^
**Hemi-arch**		
**Analysis time**	**GIC+ Petroleum jelly**	**GIC + NPC**
6 months	86 (29)^a,A^	106 (48)^a,A^
12 months	149(44)^a,A,B^	158 (56)^a,A^
24 months	176 (40)^a,B^	185 (52)^a,A^
36 months	180 (36)^a,B^	182 (32)^a,A^

Different lower cases indicate statistically significant difference between the columns (p<0.05) Different upper cases indicate statistically significant difference between the rows (p<0.05) GIC=glass ionomer cement; NPC=Nanofilled self-adhesive light-cured protective coating

## Discussion

High viscous GIC has been widely used in pediatric dentistry and considered an effective material to treat dental caries, especially within the ART approach. ^1^ ^,^ ^14^ In general, single surface restorations have shown better results than multi-surface ones ^14^ and the most relevant shortcomings of these restorations are related to the low wear resistance and brittleness of the material. ^23^ Several studies were done to simulate the wear of dental materials in the laboratory; ^6^ ^,^ ^9^ ^,^ ^10^ however, clinical studies are essential for a good understanding of the wear behavior of dental materials when submitted to the complex masticatory phenomenon that occurs in the mouth. ^12^ Given this context, we performed this investigation seeking to quantitatively evaluate the clinical wear of approximal GIC restorations in primary molars over three years. Ours results showed decreased wear of approximal GIC restorations protected with NPC when compared to restorations protected with petroleum jelly. Therefore, the hypothesis tested could be accepted.

Although the behavior of GIC protected with NPC is still not completely understood, the data available from clinical studies show a trend towards a better performance of GIC being attributed to the use of this type of protective coating. Friedl, Hiller and Friedl ^16^ (2011) reported an increased volume loss in large single- and multi-surface restorations in permanent molars after two years; however, all restorations were considered satisfactory. Hesse, et al. ^19^ (2016) observed that the survival rate of approximal restorations in primary molars was positively influenced by the application of a NPC when evaluated after three years. Diem et al., ^11^ (2014) also reported a protective effect of a NPC against wear when applied to occlusal GIC restorations in permanent molars after one and two years. Some of the mentioned studies observed changes in wear of the restorations, however, their results came from direct visual assessment of the casts obtained from these fillings. For this reason, our study can add valuable information as we evaluated the wear of coated restorations quantitively.

In our study, 32 patients were initially selected, and 20 restorations were evaluated over a three-year period. Although the study presented a sample loss higher than 30%, we applied a statistical test to verify the sample loss distribution, which was similar between the groups, minimizing the bias attributed to the loss to follow-up. The decision to include only 32 out of the 208 originally treated patients was based on the sample sizes usually selected for this type of study. Despite the lack of information on the clinical wear of GICs in the literature, a previous study investigated the wear of core-ceramic, veneers, and enamel antagonists for a three-year period by performing impressions and superimposing the images of the scanned replicas. For such, the authors selected 36 crowns from 31 patients. ^12^ Another research was performed to investigate the wear of different materials over a period of three years and although the authors included 64 restorations, they were treated with eight different materials. ^29^ One can argue that the sample size in our research was small, and that this may have compromised the external validity to a certain extent. Extrapolating data from small studies is possible, ^18^ and the results of our investigation are valuable, as they help to explain the behavior of coated GIC approximal restorations in primary molars in terms of clinical wear.

The thin layer of petroleum jelly that was applied - and carefully removed - from the restoration surface might have interfered in the bonding between the NPC and the GIC; however, we cannot precise to which extent. Regardless, our results showed that the restorations protected with NPC presented a higher wear resistance when compared to those protected only with petroleum jelly. Additionally, the results of an *in vivo* study published by our research group showed that the coated restorations presented higher survival rates when compared to those protected with petroleum jelly. ^19^ Therefore, we believe that the bonding of the coat to the GIC was sufficient to protect the material during its maturation period, thus, preserving its mechanical properties.

Although the coated restorations presented a decreased wear when compared to the restorations protected with petroleum jelly, this was only observed in the selected 1 mm^2^ area of restorations, and not when the entire hemiarches were compared. A possible explanation for this finding is that the analysis of the hemiarch considered the wear of the dental material, as well as the physiological wear of primary teeth, possibly resulting in the loss of statistical significance between the groups. Nonetheless, this factor could be interpreted as an advantage of GIC when used in primary teeth, as the material wears along with the tooth. If the wear of the dental material is smaller than the tooth structure, premature contacts could occur, resulting in fastened root resorption, ^25^ ankylosis ^4^ and functional posterior cross bite. ^8^

According to Bonifácio, de Jager and Kleverlaan ^5^ (2013) the simulated mastication stress in the GIC restorations at a minimum load may already put the entire restoration at risk of failure. Therefore, analyzing the wear of a 1 mm^2^ area of the restoration becomes important, as it represents the area where the cusps of the antagonist tooth interact with the restoration and probably represents the area where the greatest concentration of forces occur during mastication. We believe that the explanation for the decreased wear found in the GIC restorations protected with a NPC may be supported by two aspects already known: the first is the possible correlation existing between the flexural strength (FS) and the clinical wear of restorative materials, ^6^ and the second relies on the observation that this coat penetrates in the porosities commonly found in GICs. ^6^ ^,^ ^24^

The immediate mechanical properties of GIC are known to be low, and even considered insufficient to withstand mastication forces, but they increase over time. ^2^ ^,^ ^33^ ^,^ ^34^ As already mentioned, GIC is susceptible to water content changing during the setting period, which can decrease the mechanical properties of this material, ^7^ ^,^ ^22^ including the FS values. ^6^ Therefore, to improve the initial FS and avoid the decrease in other mechanical properties, protecting the material's surface is advised. ^7^ ^,^ ^22^ Although petroleum jelly is recommended as surface protection, ^7^ it can be easily washed away from the restoration surface, ^13^ resulting in a lack of GIC surface protection during the critical setting period of this material. Since the NPC used in this research is capable of micro-mechanical interlock with the GIC, ^6^ it probably lasts longer on GIC's surface. Therefore, surface protection is achieved during the entire setting reaction of GIC, resulting in an increased FS of the material ^6^ ^,^ ^24^ and a possible protection against clinical wear, which was actually observed in our study.

Given this context, the presence of surface pores and cracks in the GIC are also considered a drawback of this material, since there reports that the propagation of these cracks may result in internal fragility and reduced wear resistance, leading to restoration failures. ^24^ As previously mentioned, the NPC may promote a micro-mechanical interlock while filling the superficial cracks and pores of GIC ^6^ and we speculate that, as a consequence of the porosity reduction the propagation of cracks also decreases, possibly resulting in a higher wear resistance of the material, as confirmed by our results. Thus, the NPC could be advised as a surface protection material to be used together with GIC restorations to increase the mechanical properties of the later.

## Conclusion

In conclusion, the results of our study suggest that the application of a NPC provides protection against the clinical wear of approximal GIC restorations in primary teeth when compared to the restorations protected with petroleum jelly.
